# Risk factors for long-term mortality in patients admitted with severe infection

**DOI:** 10.1186/s12879-018-3054-4

**Published:** 2018-04-05

**Authors:** J. Francisco, I. Aragão, T. Cardoso

**Affiliations:** 1 Serviço de Medicina, Hospital de Santo António, Largo Prof. Abel Salazar, 4099-001 Porto, Portugal; 2 Unidade de Cuidados Intensivos Polivalente, Hospital de Santo António, University of Porto, Largo Prof. Abel Salazar, 4099-001 Porto, Portugal

**Keywords:** Severe infection, Risk factors for long-term mortality, 5-year mortality, ESKAPE pathogens

## Abstract

**Background:**

Severe infection is a main cause of mortality. We aim to describe risk factors for long-term mortality among inpatients with severe infection.

**Methods:**

Prospective cohort study in a 600-bed university hospital in Portugal including all patients with severe infection admitted into intensive care, medical, surgical, hematology and nephrology wards over one-year period. The outcome of interest was 5-year mortality following infection. Variables of patient background and infectious episode were studied in association with the main outcome through multiple logistic regression. There were 1013 patients included in the study. Hospital and 5-year mortality rates were 14 and 37%, respectively.

**Results:**

Two different models were developed (with and without acute-illness severity scores) and factors independently associated with 5-year mortality were [adjusted odds ratio (95% confidence interval)]: age = 1.03 per year (1.02-1.04), cancer = 4.36 (1.65–11.53), no comorbidities = 0.4 (0.26–0.62), Karnovsky Index < 70 = 2.25 (1.48–3.40), SAPS (Simplified Acute Physiology Score) II = 1.05 per point (1.03–1.07), positive blood cultures = 1.57 (1.01–2.44) and infection by an ESKAPE pathogen (*Enterococcus faecium*, *Staphylococcus aureus, Klebsiella pneumoniae, Acinetobacter baumannii, Pseudomonas aeroginosa* and *Enterobacter species*) = 1.61 (1.00– 2.60); and in the second model [without SAPS II and SOFA (Sequential Organ Failure Assessment) scores]: age = 1.04 per year (1.03–1.05), cancer = 5.93 (2.26–15.51), chronic haematologic disease = 2.37 (1.14–4.93), no comorbidities = 0.45 (0.29–0.69), Karnovsky Index< 70 = 2.32 (1.54– 3.50), septic shock [reference is infection without SIRS (Systemic Inflammatory Response Syndrome)] = 3.77 (1.80–7.89) and infection by an ESKAPE pathogen = 1.61 (1.00–2.60). Both models presented a good discrimination power with an AU-ROC curve (95% CI) of 0.81 (0.77–0.84) for model 1 and 0.80 (0.76–0.83) for model 2. If only patients that survived hospital admission are included in the model, variables retained are: age = 1.03 per year (1.02–1.05), cancer = 4.69 (1.71–12.83), chronic respiratory disease = 2.27 (1.09–4.69), diabetes mellitus = 1.65 (1.06–2.56), Karnovsky Index < 70 = 2.50 (1.63–3.83) and positive blood cultures = 1.66 (1.04–2.64) with an AU-ROC curve of 0.77 (0.73–0.81).

**Conclusions:**

Age, previous comorbidities, and functional status and infection by an ESKAPE pathogen were consistently associated with long-term prognosis. This information may help in the discussion of individual prognosis and clinical decision-making.

## Background

Severe infection is the leading cause of non-scheduled hospital admission [1]. Depending on the severity of infection mortality rate can be as high as 60% in septic shock patients [2].

But consequences of severe infection extend well beyond the first month following it, with an increased mortality during at least the first year [3].

Prognostic factors associated with sepsis are well studied by many authors, especially among the Intensive Care Unit (ICU) population, but even in this specific population they all refer to short term mortality (ICU [4], hospital [1, 5]^,^ or 28-day mortality [6]). Age [4, 7], comorbidities [2, 4–6], severity of acute illness [2, 4, 8], focus of infection [2, 4, 6, 9], place of acquisition (community, hospital or ICU-acquired) [2, 5] and infection by specific organisms [2, 4] have been nominated as potential risk factors in this sub-population.

Since many patients admitted to hospital with infection have important concomitant medical conditions that may influence long-term prognosis it seems important to study long-term mortality.

As far as we know there are no studies on long-term prognostic factors in general hospital patients with infection. The aim of the current study is to determine independent risk factors for 5-year mortality in hospitalized patients with severe infection.

## Methods

### Ethics statement

This study was approved by the Institutional Review Board of Hospital de Santo António, Oporto Hospital Centre, Portugal, and informed consent was waived due to the observational nature of the study.

### Study design and patient population

Prospective cohort study conducted at a 600-bed tertiary care university hospital, over 1-year period (1st June 2008 to 31st May 2009). All consecutive adult patients admitted to the medical, surgical, nephrology or hematology wards of the hospital or to the intensive care unit (ICU) that had a diagnosis of infection were included, based on the Center for Disease Control (CDC) criteria [10]. Infections were classified as community-acquired (CAI), healthcare-associated (HCAI) or hospital-acquired, according to the place of acquisition. Long term mortality was defined as mortality 5 years after the infection diagnosis.

Data concerning mortality was obtained from *SClinico Hospitalar*, an informatic tool connected to a national network that gathers healthcare information nationwide.

### Definitions

Severe infection was defined as an infection which led to hospital admission (with or without sepsis criteria).

CAI was defined as an infection detected within 48 h of hospital admission in patients who did not fit the criteria for a HCAI.

HCAI was defined using the same criteria of Deborah Friedman [11], an infection present at the time of hospital admission or within 48 h of admission in patients that fulfilled any of the following criteria:

- received intravenous therapy at home, wound care or specialized nursing care through a healthcare agency, family or friends; or, self-administered intravenous medical therapy in the 30 day period before the onset of the infection;
- attended a hospital or haemodialysis clinic, or received intravenous chemotherapy in the previous 30 days;
- were hospitalized in an acute care hospital for 2 or more days in the previous 90 days;
- resided in a nursing home or long-term care facility.

HAI was defined as a localized or systemic condition that resulted from an adverse reaction to the presence of an infectious agent(s) or its toxin(s), and that occurred 48 h or more after hospital admission and was not incubating at the time of admission [10]. Infections in patients recently discharged from the hospital within the previous 2-week period were also included in this group.

The CDC definitions were used to define infections at different anatomic sites [10].

We grouped *Enterococcus faecium* vancomycin-resistant, methicillin-resistant *Staphylococcus aureus* (MRSA), extended-spectrum beta-lactamase (ESBL) producer *Escherichia coli (E. coli)* and *Klebsiella* species, *Klebsiella pneumoniae* Carbapenamase-hydrolyzing and multidrug resistant (MDR) *Acinectobacter baumannii*, *Pseudomonas aeruginosa* and *Enterobacter* species in a group denominated ESKAPE [12].

The presence of ESBL production among *E. coli* and *Klebsiella* spp. strains was screened by the automatic analyzer Vitek2 (BioMérieux). It was always confirmed by a disk diffusion test that detects synergism between the cephalosporins/monobactam and clavulanate. If the interpretation of the results was doubtful we also performed Etest®: the combination strains of cefotaxime and cefotaxime/clavulanate and ceftazidime and ceftazidime/clavulanate allows the detection of ESBL whenever the ratio antibiotic/antibiotic + inhibitor is equal or above 8.

The presence of carbapenemase production in Enterobacteriaceae was suspected whenever MIC’s for ertapenem, imipenem and meropenem exceed 0.5, 1 and 1 μg/mL, respectively. In such cases, a modified Hodge test was performed, and the ultimate confirmatory test was carbapenemase detection by molecular methods.

The comorbidities studied included immunosuppression (administration of chemotherapy in the 12 months prior to hospital admission, either radiation therapy or administration of 0.2 mg/kg/day of prednisolone for at least 3 months prior to hospital admission, administration of 1 mg/kg/day of prednisolone for 1 week in the 3 months prior to hospital admission or infection with human immunodeficiency virus), chronic liver disease [13], chronic heart failure [13], chronic respiratory disease [13], hematological disease [14], cancer [14], diabetes mellitus requiring insulin therapy or oral hypoglycaemic agents before the infection and/or atherosclerosis (defined as a previous history of a transient ischemic attack, stroke, angina, myocardial infarction or peripheral arterial disease).

Functional performance status was assessed by the Karnofsky index [15]. A score of lower than 70 implies that the patient is unable to perform normal activities or do active work.

For the first day of antibiotic therapy, the acute physiological scores, The Simplified Acute Physiology Score (SAPS II) [14] and Sepsis-related Organ failure Assessment (SOFA) were recorded [16].

The initial empirical antibiotic treatment was considered “adequate” if the initial antibiotic prescribed within the 24 h matched in vitro susceptibility of a pathogen deemed to be the likely cause of infection and when the dosage and route of administration were appropriate for current medical status (focus and severity of infection); only patients with positive microbiology will be considered in this analysis.

### Statistical analysis

Continuous variables are described as means and standard deviations (SD), categorical variables are described with absolute frequencies and percentages. Student T-tests or Mann-Whitney tests are used to compare continuous values between types of infection. For categorical variables these comparisons are performed using Pearson χ^2^ test.

Variables associated with long term mortality were studied through logistic regression. Variables studied through the multiple regression logistic model were: age, functional status (Karnofsky Index), diabetes, atherosclerosis, cancer, type of infection (community, healthcare associated or hospital acquired), severity of infection, SAPS II and SOFA scores calculated for the day of infection diagnosis, site of infection, microbiological documentation of infection, positive blood cultures, infection by a multidrug resistant pathogen or an infectious agent from the group ESKAPE and inappropriate antibiotic therapy. Those with a clear association in the univariate analysis (*p*-value < 0.1) or considered clinically significant were selected for the multivariable analysis. The results of the multivariable models are expressed as odds ratio (OR) with 95% confidence interval (CI_95%_) and *p*-values. The accuracy of the models was assessed by the area under the receiver operating characteristics curve (AU-ROC) and calibration was tested using the Hosmer-Lemeshow goodness-of-fit test. The significance level was defined as *p* < 0.05.

Data were analysed using SPSS, version 18 for Windows (Chicago, IL).

## Results

There were 1035 records included in the initial study, 22 (2%) were excluded from the present analysis due to insufficient data regarding long-term outcome. Of the 1013 patients included, 86% (*n* = 868) were recruited in the ward and 14% (*n* = 145) in ICU.

Mean ± SD age of included patients was 65 ± 20 years and 51% were female (*n* = 517). Most of them, 65% (*n* = 661) had at least one comorbidity and 30% (*n* = 300) had more than one (Table 1).

**Table 1:** General characteristics patients included in the study and its association with 5 year mortality

Variable	Total	Dead at 5 years	Crude OR (95% CI)	*p* value
Age, median ± SD, years	65 ± 20	74 ± 14	1.05 (1.04–1.05), per year	< 0.001
Female, n (%)	517 (51)	190 (50)	1.06 (0.82–1.37)	0.656
Underlying conditions, n (%)				
Diabetes mellitus	198 (20)	85 (22)	1.33 (0.97–1.83)	0.074
Atherosclerosis	236 (23)	127 (34)	2.43 (1.80–3.27)	< 0.001
Immunosuppression	219 (22)	75 (20)	0.84 (0.61–1.15)	0.274
Chemotherapy	35 (4)	26 (7)		
Radiotherapy	8 (1)	6 (2)		
Long-term corticoid	168 (17)	46 (12)		
Short-term corticoid	22 (2)	11 (3)		
HIV positive (non-AIDS)	6 (1)	1 (0)		
AIDS	3 (0)	2 (1)		
Chronic liver disease	22 (2)	14 (4)	3.00 (1.25–7.22)	0.014
Chronic heart failure	74 (7)	44 (12)	2.64 (1.63–4.29)	< 0.001
Chronic respiratory disease	66 (7)	43 (11)	3.40 (2.01–5.74)	< 0.001
Chronic kidney disease	148 (15)	56 (15)	1.02 (0.71–1.46)	0.908
End Stage kidney disease	69 (7)	23 (6)	0.83 (0.49–1.39)	0.469
Cancer	45 (4)	36 (10)	7.29 (3.47–15.41)	< 0.001
Haematologic cancer	59 (6)	35 (9)	2.59 (1.51–4.42)	0.001
No comorbidities	352 (35)	78 (21)	0.34 (0.25–0.46)	< 0.001
Karnofsky Index < 70	311 (31)	195 (52)	4.73 (3.56–6.29)	< 0.001
Type of infection				< 0.001
Community	483 (48)	144 (38)	1.0	
Healthcare associated	219 (22)	106 (28)	2.21 (1.59–3.07)	
Hospital-acquired	311 (31)	129 (34)	1.67 (1.24–2.25)	
Severity of infection				< 0.001
Infection	275 (27)	86 (23)	1.0	
Sepsis	355 (35)	125 (33)	1.19 (0.85–1.67)	
Severe sepsis	292 (29)	116 (31)	1.45 (1.02–2.05)	
Septic shock	91 (9)	52 (14)	2.93 (1.80–4.77)	
SAPS II score, per point	29 ± 13	35 ± 14	1.07 (1.06–1.09)	< 0.001
SOFA score, per point	2 ± 3	3 ± 3	1.11 (1.06–1.16)	< 0.001
Focus of infection				0.043
Respiratory	407 (40)	156 (41)	1.0	
Urinary	339 (34)	134 (35)	1.05 (0.78–1.41)	
Abdominal	209 (21)	62 (16)	0.68 (0.47–0.97)	
Other	19 (2)	14 (4)	1.40 (0.81–2.43)	
Microbiologic documentation	691 (68)	276 (73)	1.41 (1.07–1.87)	0.015
Positive blood cultures	151 (15)	70 (19)	1.55 (1.09–2.19)	0.014
Infection by a MDR pathogen	322 (51)	146 (56)	1.42 (1.03–1.95)	0.031
Infection by an ESKAPE pathogen	113 (18)	61 (24)	1.88 (1.25–2.83)	0.003
Inappropriate antibiotherapy	144 (21)	73 (26)	1.74 (1.20–2.52)	0.003

*CI* Confidence interval, *SD* Standard deviation, *HIV* Human immunodeficiency virus, *AIDS* Acquired immunodeficiency syndrome, *OR* Odds ratio

The most common foci of infection were respiratory, urinary and intra-abdominal (Table 1). Overall isolation rate was 68% (*n* = 691) (Table 2). Initial antibiotic therapy was inadequate in 18% (*n* = 179) of the patients included.

**Table 2:** Isolated infectious agents

Isolated microorganisms, n (%)	Total	Dead at 5 years
Community-acquired infection	272 (56)	93 (65)
*Escherichia coli*	101 (10)	39 (10)
*Streptococcus pneumoniae*	59 (6)	18 (5)
*Haemophilus influenza*	17 (2)	3 (1)
*Proteus mirabillis*	12 (1)	5 (1)
*Klebsiella pneumoniae*	11 (1)	5 (1)
*Pseudomonas aeroginosa*	10 (10)	5 (1)
*Enterococcus faecium*	7 (0)	4 (1)
MSSA	7 (0)	2 (1)
*Legionella pneumophyla*	6 (0)	0 (0)
Other	42 (4)	21 (6)
ESKAPE	10 (10)	5 (1)
MDR	79 (8)	36 (10)
Healthcare-associated infection	160 (73)	76 (72)
*Escherichia coli*	69 (7)	29 (8)
MSSA	21 (2)	9 (2)
*Klebsiella pneumoniae*	15 (2)	6 (2)
*Pseudomonas aeroginosa*	11 (1)	6 (2)
*Enterococcus faecalis*	9 (0)	4 (1)
MRSA	8 (0)	6 (2)
*Proteus mirabillis*	7 (0)	4 (1)
*Streptococcus pneumoniae*	7 (0)	5 (1)
*Enterococcus faecium*	6 (0)	4 (1)
Other	31 (3)	18 (5)
ESKAPE	30 (3)	16 (4)
MDR	85 (8)	44 (12)
Hospital-acquired infection	259 (833)	107 (83)
*Escherichia coli*	68 (7)	24 (6)
*Pseudomonas aeroginosa*	37 (4)	18 (2)
MRSA	30 (3)	18 (5)
*Enterococcus faecalis*	24 (2)	9 (2)
*Klebsiella pneumoniae*	23 (2)	7 (2)
MSSA	18 (2)	3 (1)
*Proteus mirabillis*	17 (2)	8 (2)
*Enterobacter cloacae*	16 (2)	8 (0)
*Acinetobacter baumanni*	13 (1)	4 (0)
*Enterococcus faecium*	13 (1)	7 (2)
*Candida albicans*	7 (0)	0 (0)
*Morganella morganni*	6 (0)	1 (0)
*Clostridium difficile*	5 (0)	3 (1)
*Enterobacter aerogenes*	5 (0)	2 (1)
*Serratia marcescens*	5 (0)	3 (1)
Other	24 (2)	14 (4)
ESKAPE	73 (7)	10 (3)
MDR	158 (16)	79 (21)

*MSSA* Methicillin-sensitive *Staphylococcus aureus*, *MRSA* Methicillin-resistant *Staphylococcus aureus*, *ESKAPE Enterococcus faecium,* MRSA*,* ESBL *Klebsiella pneumoniae, Acinetobacter baumannii, Pseudomonas aeruginosa* and *Enterobacter* species; *MDR* Multidrug resistant bactéria

Severity of acute illness was documented by a median ± SD SAPS II and SOFA scores of 30 ± 13 and 2 ± 3, respectively. Hospital and 5-year mortality rates were 14% (*n* = 137) and 37% (*n* = 379), respectively.

**Fig. 1:**
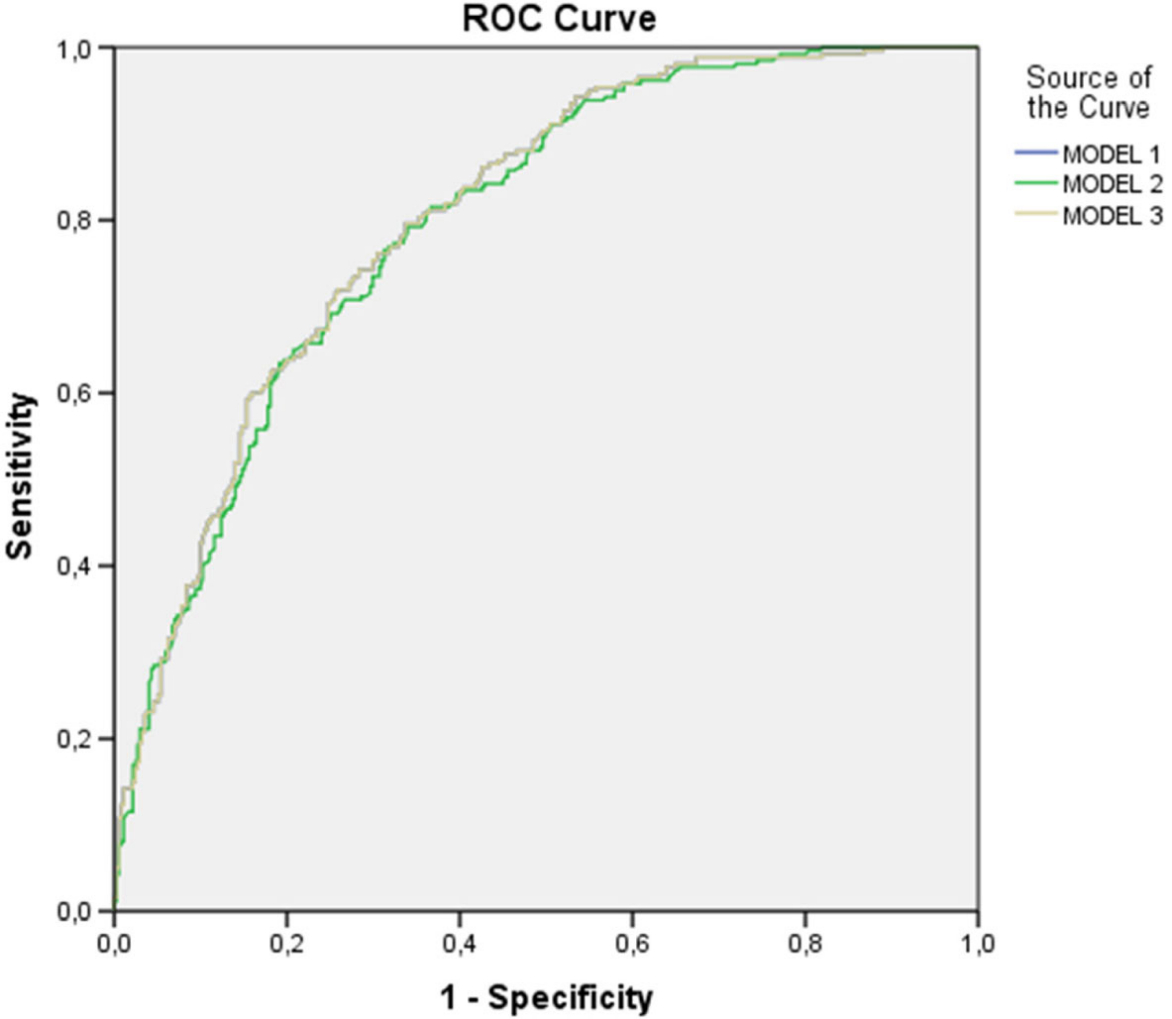
Area under the receiver operating characteristics (AU-ROC) curve (95% CI) for the final models. Model 1: all patients with acute severity scores (SPAS II and SOFA): 0.81 (0.77–0.84). Model 2: all patients without acute severity scores: 0.80 (0.76–0.83). Model 3: only patients that survived hospital admission: 0.77 (0.73–0.81)

Variables associated with 5-year mortality in the univariate analysis were: age, the presence of comorbidities, namely: diabetes, atherosclerosis, chronic liver disease, chronic heart failure, chronic respiratory failure, solid tumours and haematologic cancer, Karnofsky index< 70, type of infection, severity of infection, SAPS II and SOFA scores, focus of infection, microbiologic documentation of infection, positive blood cultures, infection by a MDR pathogen or a pathogen from the group ESKAPE and inappropriate initial antibiotic therapy (Table 1). The final model retained: age, cancer, absence of known comorbidities, Karnofsky index < 70, SAPS II, positive blood cultures and infection by a pathogen from the ESKAPE group (Model 1, Table 3). A second model without SAPS II and SOFA scores was built and the same variables were retained plus haematologic disease and severity of infection (Model 2, Table 3). The AU-ROC curve was 0.81 (0.77-0.84) and 0.80 (0.76-0.83) for the first and second models, respectively (Fig. 1).

**Table 3:** Independent risk factors associated with long term death in patients admitted with severe infection

Variable	Total	Dead at 5 years	Adjusted OR (95% CI)	
			Model 1	Model 2
Age, mean ± SD, per year	65 ± 20	74 ± 14	1,03 (1.02–1.04)	1,04 (1.03–1.05)
Cancer, n (%)	45 (4)	36 (10)	4.36 (1.65– 11.53)	5.93 (2.26– 15.51)
Chronic haematologic disease, n (%)	59 (6)	35 (9)		2.37 (1.14–4.93)
No comorbidities, n (%)	352 (35)	78 (21)	0.40 (0.26–0.62)	0.45 (0.29–0.69)
Karnofsky Index < 70, n (%)	311 (31)	195 (52)	2.25 (1.48–3.40)	2.32 (1.54–3.50)
Severity of infection, n (%)				
Infection	275 (27)	86 (23)		1.00
Sepsis	355 (35)	125 (33)		1.15 (0.72–1.83)
Severe sepsis	292 (29)	116 (31)		1.33 (0.81– 2.17)
Septic shock	91 (9)	52 (14)		3.77 (1.80– 7.89)
SAPS II, median ± SD, per point	29 ± 13	35 ± 14	1.05 (1.03–1.07)	
Positive blood cultures, n (%)	151 (15)	70 (19)	1.57 (1.01–2.44)	
Infection by an ESKAPE pathogen, n (%)	113 (18)	61 (24)	1.61 (1.00–2.60)	1.61 (1.00–2.60)

*OR* Odds ratio, *CI* Confidence interval, *ESKAPE Enterococcus faecium, Staphylococcus aureus, Klebsiella pneumoniae, Acinetobacter baumannii, Pseudomonas aeruginosa* and *Enterobacter* species

A third model considering only patients that were discharged alive from the hospital was made and results are shown in Table 4. The variables retained were: age, comorbidities (cancer, chronic respiratory disease and *diabetes mellitus*), karnofsky index< 70 and positive blood cultures (Table 4). The AU-ROC curve was 0.77 (0.73-0.81) (Fig. 1).

**Table 4:** Characteristics of the group of patients discharged alive and its association with 5 year mortality

Variable	Total (*n* = 876)	Dead at 5 years (*n* = 243)	Crude OR (95% CI)	*p* value	Adjusted OR (95% CI)
Age, median ± SD, years	63 ± 20	73 ± 14	1.04 (1.03–1.05), per year	< 0.001	1.03, per year (1.02–1.05)
Female, n (%)	458 (52)	132 (54)	0.89 (0.66–1.20)	0.454	
Underlying conditions, n (%)					
Diabetes mellitus	183 (21)	70 (29)	1.86 (1.32–2.63)	< 0.001	1.65 (1.06–2.56)
Atherosclerosis	283 (21)	74 (31)	2.11 (1.50–2.96)	< 0.001	
Immunosuppression	195 (22)	51 (21)	0.90 (0.63–1.29)	0.575	
Chemotherapy	26 (3)	17 (7)			
Radiotherapy	6 (1)	4 (2)			
Long-term corticoid	166 (18)	34 (14)			
Short-term corticoid	16 (2)	5 (2)			
HIV positive (non-AIDS)	6 (1)	1 (0)			
AIDS	2 (0)	1 (0)			
Chronic liver disease	14 (2)	6 (3)	1.98 (0.68–5.76)	0.211	
Chronic heart failure	62 (7)	33 (14)	3.27 (1.94–5.52)	< 0.001	
Chronic respiratory disease	66 (7)	43 (11,4)	4.17 (2.39–7.26)	< 0.001	2.27 (1.09–4.69)
Chronic kidney disease	138 (16)	46 (19)	1.37 (0.93–2.03)	0.111	
End Stage kidney disease	66 (8)	20 (8)	1.14 (0.66–1.98)	0.639	
Cancer	27 (3)	18 (7)	5.55 (2.46–12.53)	< 0.001	4.69 (1.71–12.83)
Haematologic cancer	42 (5)	18 (7)	2.03 (1.08–3.81)	0.028	
No comorbidities	322 (37)	48 (20)	0.32 (0.23–0.46)	< 0.001	
Karnofsky Index < 70	230 (26)	115 (47)	4.05 (2.93–5.59)	< 0.001	2.50 (1.63–3.83)
Type of infection				< 0.001	
Community	436 (50)	97 (40)	1.0		
Healthcare associated	187 (21)	75 (31)	2.34 (1.62–3.39)		
Hospital-acquired	253 (29)	71 (29)	1.36 (0.96–1.95)		
Severity of infection				0.61	
Infection	255 (29)	66 (27)	1.0		
Sepsis	325 (37)	96 (40)	1.20 (0.83–1.73)		
Severe sepsis	246 (28)	70 (29)	1.14 (0.77–1.67)		
Septic shock	50 (6)	11 (5)	0.80 (0.39–1.67)		
SAPS II score, per point	27 ± 10	31 ± 9	1.05 (1.03–1.06)	< 0.001	
SOFA score, per point	2 ± 2	2 ± 2	0.99 (0.93–1.06)	0.771	
Focus of infection				0.005	
Respiratory	345 (39)	94 (39)	1.0		
Urinary	304 (35)	100 (41)	1.31 (0.94–1.83)		
Abdominal	280 (21)	33 (14)	0.60 (0.38–0.94)		
Other	47 (5)	16 (7)	1.38 (0.72–2.64)		
Microbiologic documentation	593 (68)	179 (74)	1.48 (1.07–2.06)	0.020	
Positive blood cultures	126 (14)	46 (19)	1.61 (1.09–2.40)	0.018	1.66 (1.04–2.64)
Infection by a MDR pathogen	265 (49)	89 (53)	1.24 (0.86–1.79)	0.245	
Infection by an ESKAPE pathogen	82 (15)	30 (18)	1.33 (0.81–2.17)	0.256	
Inappropriate antibiotherapy	113 (19)	43 (24)	1.55 (1.01–2.39)	0.044	

*CI* Confidence interval, *SD* Standard deviation, *HIV* Human immunodeficiency virus, *AIDS* Acquired immunodeficiency syndrome, *OR* Odds ratio

## Discussion

The 5-year mortality rate in our cohort was 37%. Previous articles have described a 5-year mortality between 39 and 74% [17–21]. This difference could be explained partially by the implementation of the *Surviving Sepsis Campaign* in 2004 that resulted in a consistent decrease in mortality due to severe infection/sepsis [22].

Hospital mortality rate was 14%, lower than described by previous authors that considered only patients admitted into ICU [2].

In general, predictors of long-term mortality found in this study are similar to those from other studies, like: age [20, 23–27], comorbidities [19, 20, 23–26], functional status [23], severity of infection [20, 23], SAPS II, positive blood cultures and infection by an ESKAPE pathogen. The association of an infection by a pathogen from the ESKAPE group with long-term mortality has not been described previously, as far as the authors are aware.

One surprising result was the fact that inappropriate antibiotic therapy was not retained as an independent prognostic factor, although this was shown to influence long-term mortality following bacteraemia [20, 23], but given the high proportion of patients that received appropriate antibiotic therapy in the first 24 h its impact in this cohort may be less evident.

In patients that survived hospital admission the infection-related risk factors were less significant, aside from positive blood cultures, and patient related factors were more relevant like age, comorbidities and functional status.

In the acute setting it is reasonable to expect infection to be the dominant cause of death. However in long-term mortality infection may play less of a direct role. Maybe it can be due to a combination of pre-existing co-morbidities, intensities of therapy (and its iatrogenic effects) the nature and severity of initial infection and the complications of the acute disease. Therefore the mechanism by which certain risk factors independently affect long-term prognosis should be investigated [17]. Another important question is whether prevention or optimal management of these parallel conditions might reduce the long-term death rates [19]. If post-sepsis long-term outcomes are primarily driven by the trajectory of pre-morbid conditions, then interventions targeted at complications attributed to critical illness may not be effective [28]. However all risk factors related to long-term mortality should be considered when addressing individual prognosis and making clinical decision.

This study has several limitations. It is a single center study and although the study design was prospective, data regarding 5 year outcome was collected retrospectively, leading to the exclusion of a minority of patients; nevertheless the final database was of very good quality [29]. We did not have a control population (general population or non-infected sample) to determine the true impact of severe infection. We did not collect data on the ultimate cause of death which would have been very important to identify modifiable prognostic factors that could improve long term outcomes. Secondly, we defined long-term mortality being a 5-year period as there is no consensus towards the definition of long-term outcomes. Finally, we have only studied one long-term outcome leaving others behind (namely those related to quality of life).

Our study has also several strengths; it includes a large cohort of patients, from different hospital settings, with different focus of infection. Previous studies have been restricted to intensive care patients [2, 4–9], specific focus of infection [20, 25, 27] or specific pathogens [21, 24].

## Conclusions

Age, cancer, comorbidities, functional status (Karnovsky Index < 70), SAPS II, severity of infection, positive blood cultures, and infection by a pathogen from the ESKAPE group were independently associated with increased 5-year mortality in this large group of patients with severe infection.

We hope that this information will help in the discussion of individual prognosis and clinical decision making.

## Abbreviations

AU-ROC: Area under the receiver operating characteristics
CAI: Community-acquired infection
CDC: Center for Disease Control
*E. coli*: *Escherichia coli*
ESBL: Extended-spectrum beta-lactamase
ESKAPE: *Enterococcus faecium,* MRSA*,* ESBL *Klebsiella pneumoniae, Acinetobacter baumannii, Pseudomonas aeruginosa* and *Enterobacter* species;
HAI: Hospital-acquired infection
HCAI: Healthcare-associated infections
ICU: Intensive Care Unit
MDR: Multidrug resistant
MIC: Minimum inhibitory concentration
MRSA: Methicillin-resistant *Staphylococcus aureus*;
MSSA: Methicillin-sensitive *Staphylococcus aureus*
OR: Odds ratio
SD: Standard deviations
